# Is the New Variant RHDV Replacing Genogroup 1 in Portuguese Wild Rabbit Populations?

**DOI:** 10.3390/v7010027

**Published:** 2014-12-30

**Authors:** Ana M. Lopes, Jorge Correia, Joana Abrantes, Pedro Melo, Margarida Ramada, Maria J. Magalhães, Paulo C. Alves, Pedro J. Esteves

**Affiliations:** 1 CIBIO, InBIO—Research Network in Biodiversity and Evolutionary Biology, Universidade do Porto, Campus de Vairão, Rua Padre Armando Quintas, 4485-661 Vairão, Portugal; E-Mails: analopes@cibio.up.pt (A.M.L.); jabrantes@cibio.up.pt (J.A.); magalhaes.mjtr@hotmail.com (M.J.M.); pcalves@fc.up.pt (P.C.A.); 2 Departamento de Biologia, Faculdade de Ciências da Universidade do Porto, Rua do Campo Alegre, s/n, 4169-007, Porto, Portugal; 3 INSERM—Institut national de la santé et de la recherche médicale, U892, Université de Nantes, 44007 Nantes, France; 4 CIISA—Centro de Investigação Interdisciplinar em Sanidade Animal, Faculdade de Medicina Veterinária, Universidade de Lisboa, 1300-477 Lisboa, Portugal; E-Mail: jcorreia@fmv.ulisboa.pt; 5 VETNATURA, 1600-176 Lisboa, Portugal; E-Mails: vetnatura@gmail.com (P.M.); margarida.ramada@dcv.eu.com (M.R.); 6 Wildlife Biology Program, University of Montana, 32 Campus Drive, Missoula, MT 59812, USA; 7 CITS—Centro de Investigação em Tecnologias da Saúde, IPSN, CESPU, 4585-116 Gandra, Portugal

**Keywords:** rabbit hemorrhagic disease virus (RHDV), rabbit hemorrhagic disease (RHD), new variant RHDV, genogroup 1, replacement, Portugal

## Abstract

The *Lagovirus* rabbit hemorrhagic disease virus (RHDV), a member of the family *Caliciviridae*, severely affects European rabbit (*Oryctolagus cuniculus*) populations by causing rabbit hemorrhagic disease (RHD). RHDV is subdivided in six genogroups but, more recently, a new RHDV variant with a unique genetic and antigenic profile emerged. We performed a study in rabbits found dead in the field during 2013 and 2014 in Portugal to determine the prevalence of this new variant *versus* the classical RHDV. Fifty-seven liver samples were screened for the presence of RHDV and positive samples were genotyped. All cases of RHDV infection were caused by the new variant. The only former genogroup circulating in Portugal, G1, was not detected. We hence conclude that the new RHDV variant is replacing G1 in Portugal, probably due to a selective advantage. This sudden and rapid replacement emphasizes the necessity of continued monitoring of wild rabbit populations.

## 1. Introduction

Rabbit hemorrhagic disease virus (RHDV), a member of the genus *Lagovirus*, family *Caliciviridae*, is responsible for causing rabbit hemorrhagic disease (RHD). RHD was first reported in China in 1984 [1] and is a highly contagious and fatal disease for European rabbit (*Oryctolagus cuniculus*). The acute form of the disease can cause up to 80%–100% of mortality within 1–3 days [2,3]. RHD is associated with hepatic necrosis, hemorrhages and congestion in several organs, including the upper respiratory tract and lungs, and splenomegaly [2]. The disease became endemic in several European countries, where it was first reported in 1986 [4], and in Australia and New Zealand, where it was introduced as biological control agent [5]. In Portugal, RHD was described in 1989 (reviewed in [3]).

RHDV is a non-enveloped, positive-sense, single-stranded RNA virus, with a genome of ~7.4 kb organized in two ORFs, with ORF1 encoding the major structural capsid protein gene, VP60. Historically, six RHDV genogroups were recognized based on the VP60: genogroups 1–5 (G1–G5) and the antigenic variant RHDVa or G6 [6,7]. However, more recent studies showed that G3, G4 and G5 belong to the same genetic cluster [8,9]. Furthermore, weak pathogenic and non-pathogenic forms of the virus exist, which are genetically different from the pathogenic RHDV, exhibiting ~20% divergence [10,11,12,13,14]. The other member of the genus *Lagovirus*, the closely related European brown hare syndrome virus (EBHSV), has a similar organization of the genome, albeit presenting ~30% divergence from RHDV [15].

In 2010, a new RHDV variant emerged in France [16], tentatively named RHDV2 or RHDVb, that rapidly spread to several European countries including Portugal, where it was described in 2012 [17,18,19,20,21]. With a unique antigenic profile, it causes mortality in young rabbits (less than two months) and vaccinated rabbits, that are typically not susceptible to G1–G6 strains [17]. Vaccinated rabbits appear to be less susceptible to this new variant, albeit protection conferred by the vaccine is not complete [19]. Before the emergence of the new variant, only G1 strains were known to circulate in Portugal [22,23,24]. RHDVa was also described in the Iberian Peninsula [25], but this description refers only to domestic rabbits and no reported cases exist in wild animals.

The Iberian Peninsula is inhabited by two subspecies of *O. cuniculus*: one occupying the northeastern part (*O. c. cuniculus*) and the other restricted to the southwest and the islands of Madeira, Azores and Canaries (*O. c. algirus*) [reviewed in 26]. Both subspecies are equally affected by RHDV [22], but the susceptibility of the subspecies *O. c. algirus* acquires special importance since its distribution is restricted to the Iberian Peninsula. Thus, this subspecies is more vulnerable to severe outbreaks, which might have implications on its conservation status.

In this work, we performed a genetic study to determine the prevalence of the new variant comparing to G1 in Portuguese rabbit populations and a histopathological study to characterize the lesions found in young rabbits. Our results confirm that the new variant has replaced G1 in Portugal in the last two years, possibly reflecting a selective advantage.

## 2. Materials and Methods

**Figure 1:**
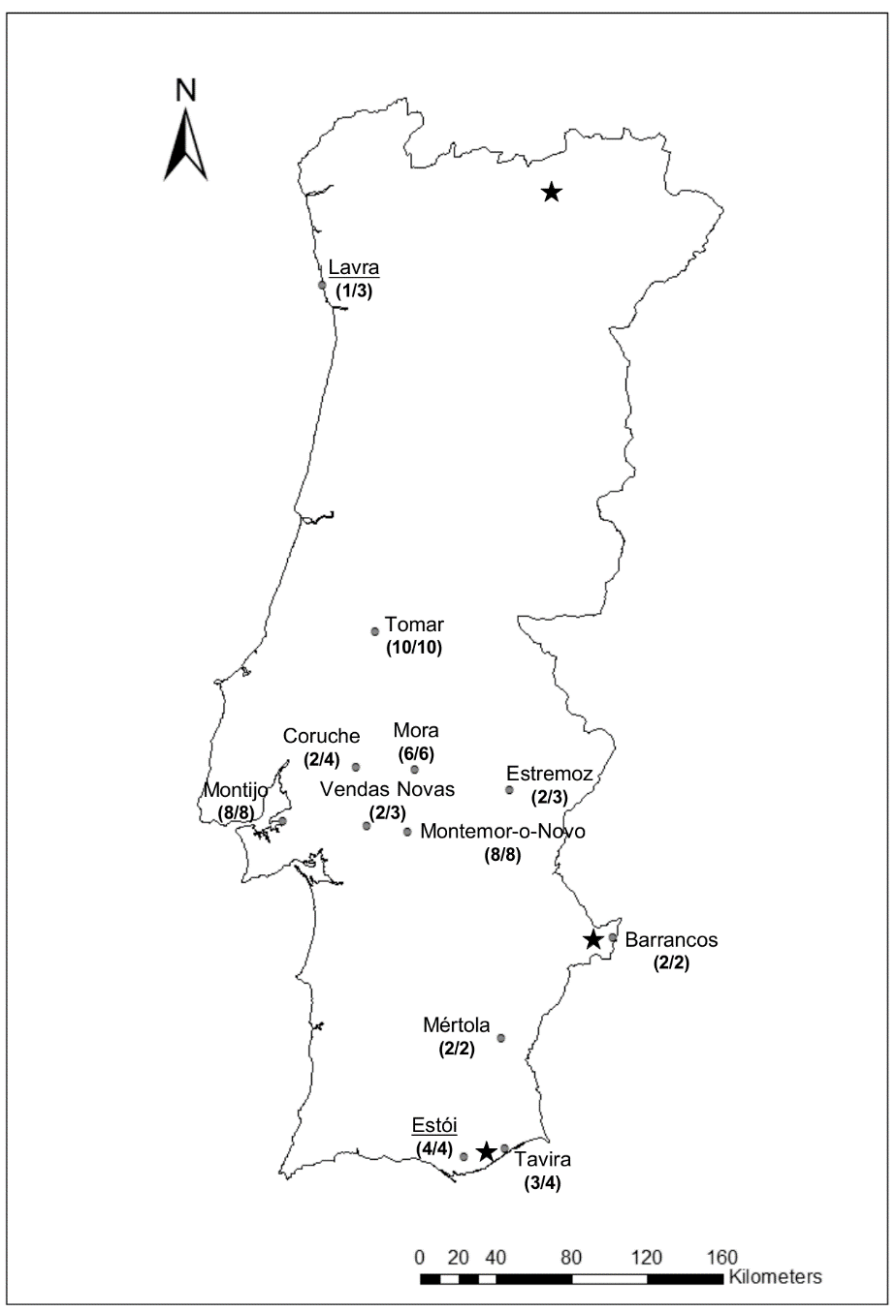
Map of Portugal with sampling localities indicated. Localities sampled in 2013 appear underlined; in parenthesis, the number of positive samples per total number of samples is shown. The approximate locations of the first descriptions of the new variant’s presence in Portugal according to [18] are marked by the black stars.

Fifty-seven liver samples were collected in 12 localities covering from north to south of Portugal, between 2013 and 2014 (Figure 1), and frozen at −20 °C. All rabbits were found dead in the field with lesions consistent with RHD and, based on the geographical distribution, belong to the subspecies *O. c. algirus* [26]. In addition, for seven young wild rabbits, carcasses were submitted to necropsy and samples of lung, liver, thymus, trachea, kidney and spleen were collected for histopathological exam. Tissue samples were fixed in 10% buffered formalin and embedded in paraffin. Sections of 3 µm thick were stained with haematoxylin and eosin for routine microscopical examination.

Total RNA was extracted from the liver of all samples with the RNeasy Mini Kit (Qiagen, Hilden, Germany). Up to 30 mg of liver were collected and tissue was homogenized in a rotor-stator homogenizer (Mixer Mill MM400 from Retsch, Haan, Germany) at 30 Hz for 7 min. RNA extraction followed the manufacturer’s instructions. Viral cDNA was synthesized using oligo(dT) primers and SuperScriptTM III Reverse Transcriptase (Invitrogen, Carlsbad, CA, USA) following the manufacturer’s instructions. A screening to test for the presence of RHDV was performed with two pairs of primers: the first pair is specific for the new variant: RHNavF 5'-GTTCGTCAAATGTACTTGAGC-3' and RHNavR 5'-GTGTACGTAATGGCACTACTG-3' [27]. Samples that tested negative were screened with a second more general pair of primers (RHDV4831F 5'-GTGTATGCCATGACTCCGAT-3' and RHDV_VP60_0467R 5'-GCGTCGATGACAACATGAG-3'). These primers were used in previous studies and successfully amplified several RHDV strains including G1, RHDVa and G3–G5 like [24,25,28,29]. For positive samples, the complete capsid protein gene, VP60, with 1740 base pairs, was amplified (PCR primers and conditions are available upon request). After purification, PCR products were sequenced on an automatic sequencer ABI PRISM 310 Genetic Analyzer (PE Applied Biosystems, Foster City, CA, USA) with the amplification primers. These sequences were deposited in GenBank with the accession numbers KM115667-KM115716 and aligned in BioEdit version 7.0.9.0 [30]. Each sequence was compared and blasted against publicly available sequences in NBCI (http://blast.ncbi.nlm.nih.gov/Blast.cgi). In order to facilitate visualization and the subsequent analyses, a maximum of ten sequences of the main RHDV groups were used (G1–G6, the new variant, non-pathogenic RCV-A1 and weakly pathogenic MRCV; EBHSV was also included). Genetic distances between our samples and each RHDV group were calculated in MEGA6 (*p*-distance method, 1000 replicates) [31]. Additionally, a Maximum Likelihood (ML) tree, using a representative of each unique haplotype found, was estimated using the same software. The best-fit nucleotide substitution model as determined in MEGA6 (GTR+G+I) and a bootstrap resampling analysis of 1000 replicates, were used to reconstruct the phylogeny.

## 3. Results and Discussion

Between 2013 and 2014, Portuguese wild rabbit populations suffered severe RHD outbreaks. Indeed, during that period, we received in our laboratory rabbit samples from several Portuguese localities (Figure 1) that were shown to be positive for the new RHDV variant. This new variant was described in Portugal in 2012 [18]. Interestingly, before that, G1 was the only genogroup known to be circulating in Portugal [22,23,24].

**Figure 2:**
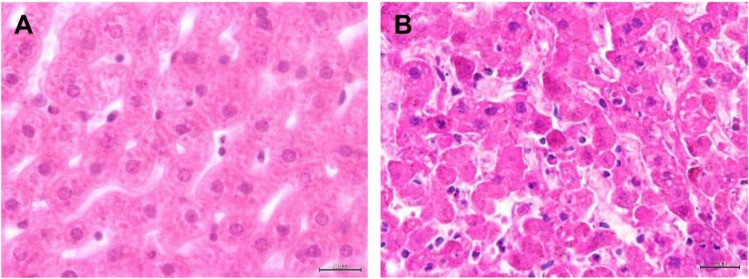
Histopathological characterization of the new rabbit hemorrhagic disease virus (RHDV) variant in Portuguese samples. (**A**) Liver section of a healthy rabbit. Staining H&E, scale bar = 20 µm; **(B)** Liver with lesions of microvacuolar degeneration and necrotizing hepatitis with karyolysis. Staining H&E, scale bar = 20 µm.

While the macroscopic and histopathological alterations caused by RHDV are well-known, little is known on how the new RHDV variant affects wild animals. As this is a new and emergent virus, monitoring these alterations might give insights on eventual changes in lesions. At necropsy, the alterations and histopathological lesions observed were similar to those observed for other animals infected with the new RHDV variant and that are typical of a RHDV infection [16,17,19]. Necropsy of the seven young rabbits revealed epistaxis, hemorrhagic tracheitis with sero-hemorrhagic exudate in the lumen of the trachea, sero-hemorrhagic pleurisy, pulmonary congestion (predominantly in the dorsal region), and multiple hemorrhages scattered throughout the organs. Livers were predominantly pale in appearance and in some cases were congested. The thymus, spleen and kidneys showed also congestion. Histopathology revealed hemorrhagic pneumonia and tracheitis, congestion of the liver and diffuse necrotizing hepatitis (Figure 2A,B). This first establishment of a diagnosis and consequent cause of death was then confirmed by the molecular techniques.

**Figure 3:**
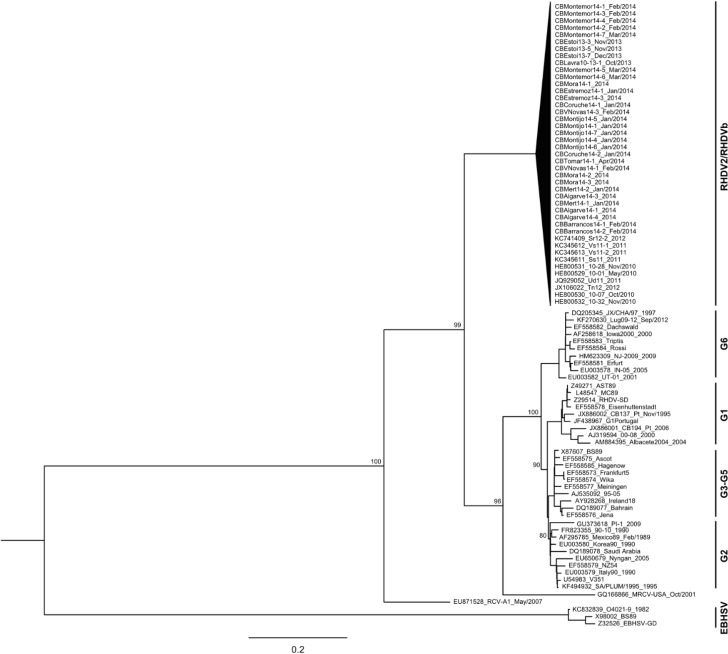
Maximum Likelihood tree of the capsid gene. Bootstrap values of the main nodes (≥0.80) are shown next to them. To facilitate visualization, the new variant group is collapsed (previously identified new variant strains have their accession numbers indicated). Samples were named according to the sampling locality, the year of collection and the number of the isolate (cf. Figure 1); when known, month and year of collection are indicated. Scale bar indicates the nucleotide substitutions per site. GenBank accession numbers of the publicly available sequences used in this study are indicated.

Of the 57 rabbit liver samples, 50 were positive for the new variant (87.7%; Figure 1); all but one were successfully amplified for the entire capsid gene. The 143 nucleotide differences defined 33 unique haplotypes, with an overall identity of 98.7% (±0.1%). None of the samples was positive for G1 or for any other genogroup. The blast analysis revealed 96%–98% of identity with publicly available sequences of the new variant from Italy and France (GenBank accession numbers KC741409, HE800529-32, FR819781, HE819400, JQ929052, KC345611-13, JX106022-23, KC907712), and an overall identity of 82% with G1 strains. The analysis of the nucleotide identities between our dataset and genogroups showed an identity of 81.6% (± 0.8%) with G2, 82.1% (± 0.8%) with RHDVa and 82.2% (± 0.8%) with G3-G5. Furthermore, our sequences presented a nucleotide identity of 81% (± 1.0%) with the weakly pathogenic MRCV, 79.2% (± 0.8%) with the non-pathogenic RCV-A1 (strain MIC-07) and 70.0% (± 0.9%) with EBHSV (overall identity with strains O4021-9, BS89 and EBHSV-GD; GenBank accession numbers of these sequences are indicated in Figure 3). The ML tree also confirmed that all samples belong to the new RHDV variant group (Figure 3), since all RHDV samples recovered from Portuguese rabbits cluster with publicly available sequences described as belonging to the new RHDV variant (bootstrap value of 99; Figure 3). Comparison with the partial sequences of the capsid obtained in the first reported cases of the new RHDV variant in Portugal (GenBank accession numbers KF442960-64) [18], revealed that our sequences present between 0.0% (± 0.2%) and 2.5% (± 0.6%) divergence. However, the small length of the analyzed fragment does not allow us to make any conclusions about the epidemiology and dispersal of the virus since the initial outbreaks.

Indeed, the lack of epidemiological data on RHDV prevalence in Portuguese wild rabbit populations impairs a precise comparison between G1 and the new variant virulence/pathogenicity. However, the number of dead animals found in the field in 2013 and 2014 seems to have substantially increased, as has been described in Spain by Delibes-Mateos et al [32]. Our results show that these RHD outbreaks were caused by the new variant and no apparent cases of G1 (or other genogroup) were detected. This indicates that the new variant is the main variant currently circulating in Portugal and has potentially replaced G1; the factors contributing to this replacement are still unclear, but are currently under study. A similar situation has been described in France and Spain, where the new RHDV variant appears to be replacing G5 and G1, respectively [19,33,34].

After the first description of the new variant in Portugal in three Portuguese localities in 2012 and 2013 [18], the new RHDV variant was able to disperse to the center and south of Portugal (Figure 1), suggesting a high capacity of dispersal in a very short period of time. The weak dispersal to the north of Portugal is possibly due to low rabbit densities. By contrast, in the south, the high densities of rabbits facilitate virus dispersal. The high capacity of dispersal, associated with the replacement of G1, suggests a selective advantage of the new variant over other genogroups, possibly by overcoming existing immunity to older strains. Le Gall-Reculé and colleagues (2013) reported that the new RHDV variant is less virulent than classical RHDV in experimental conditions and that this could be advantageous for the new variant, by giving more time to the virus to spread. The different mortality rates observed between studies (e.g., [17,19]) make possible another scenario, where the new variant is still evolving to acquire its optimal virulence. Another possibility is that the new RHDV variant might be evolving towards higher levels of virulence, as observed in a recent study in Australia, where RHDV virulence has been increasing due to development of genetic resistance in rabbits in Australia [35].

The threat presented by the new RHDV variant to the European rabbit might be particularly high for *O. c. algirus*, since distribution of this subspecies is limited to the southwest of Iberian Peninsula. In addition, the presence and circulation of the new RHDV variant compromises the reintroduction of the most endangered felid in the world (Iberian lynx, *Lynx pardinus*) in Portugal, as rabbits represent 90% of its diet. The European rabbit is also economically important as game species, being a traditionally coveted prey, with high social value. Since the epidemiological outcome of G1 replacement by the new variant is unpredictable, and the new variant is causing important ecological and economical losses in Portugal, rabbit translocations should also be highly restricted, and continued monitoring of natural rabbit populations is strongly recommended.
